# Supplementary material for: Cabotegravir Exposure of Zebrafish (Danio rerio) Embryos Impacts on Neurodevelopment and Behavior
Source: Int J Mol Sci. 2023 Jan 19;24(3):1994. doi: 10.3390/ijms24031994 (PMC9916638; doi:10.3390/ijms24031994)
Supplement: Supplementary file 1 [file ijms-24-01994-s001.zip › ijms-2145201-supplementary.pdf]

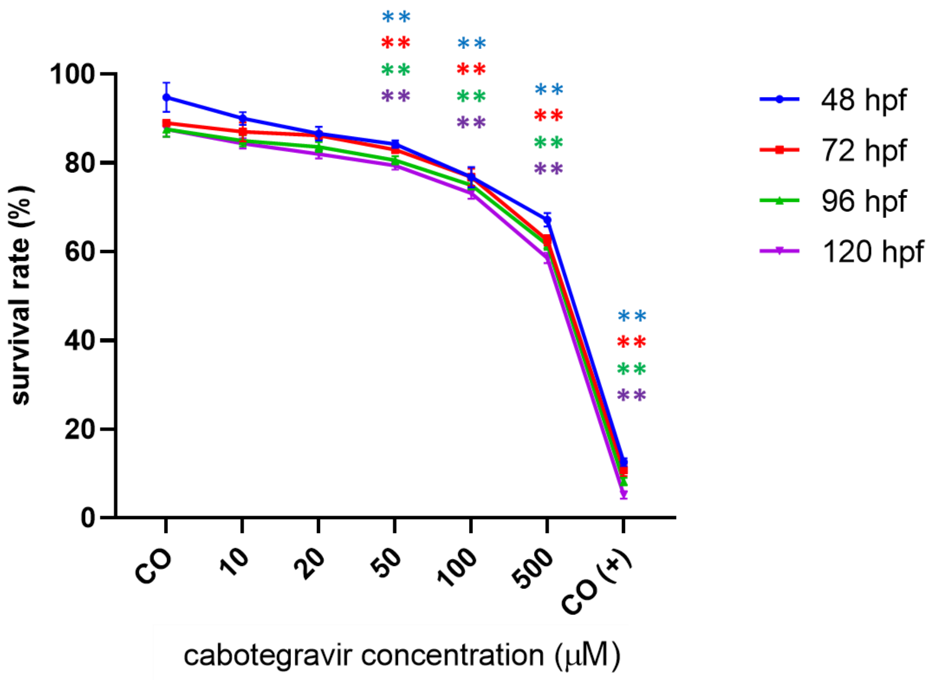

Supplementary Figure S1. Survival rate of zebrafish embryos at different developmental stages (hours post fertilization, hpf) after exposure to cabotegravir (CAB) by the immersion method. Dechorionated zebrafish embryos were exposed at gastrula stage (4 hpf) to drug solvent only (fish water plus 0.1% dimethyl sulfoxide, DMSO) [CO], 3,4-dichloroaniline (DCA) dissolved in fish water at the concentration of 3.74 mg/L as positive control [CO (+)] or CAB, dissolved in fish water containing 0.1% DMSO. X-axis shows drug doses used for exposure of embryos; Y-axis shows survival percentages at the different developmental stages (48 hpf, blue line; 72 hpf, red line; 96 hpf, green line; 120 hpf, violet line). Results are expressed as mean ± SD of three independent experiments, with 30 embryos for each experiment and each treatment. (* $p < 0.05$ vs. control group; ** $p < 0.005$ vs. control group).

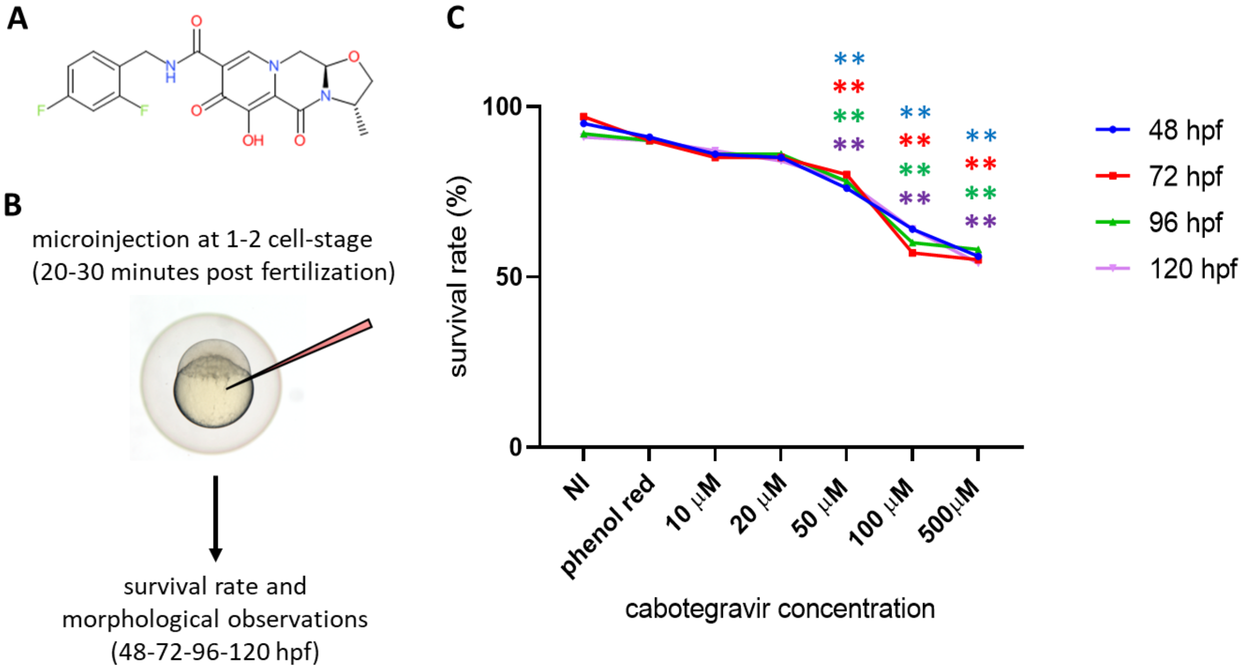

Supplementary Figure S2. Survival rate of zebrafish embryos at different developmental stages (48, 72, 96 and 120 hours post fertilization, hpf) after exposure to cabotegravir (CAB) directly microinjected at 1-2 cell stage with drug solutions. *(A)* CAB chemical structure. *(B)* Overall experimental design. *(C)* Zebrafish embryos were directly microinjected with fish water plus 0.1% DMSO and 0.05% phenol red [phenol red] as negative control or CAB [10-20-50-100-500 µM], dissolved in fish water containing 0.1% DMSO. CAB solutions were co-injected with 0.05% phenol red as a tracer and control for microinjection process. Not injected embryos were also used as further control [NI]. Total volume of injection was 5 nL/embryo. X-axis shows drug doses used for microinjection of embryos; Y-axis shows the corresponding survival percentages at 96 hpf (green line) and 120 hpf (violet line). Results are expressed as mean ± SD of three independent experiments, with 50 embryos microinjected for each experiment and each treatment. Statistical analysis was performed with GraphPad Prism 8.3 Software by Student's t test. *p*-values less than 0.05 were considered as significant (* $p < 0.05$ vs. control group; ** $p < 0.05$ vs. control group).

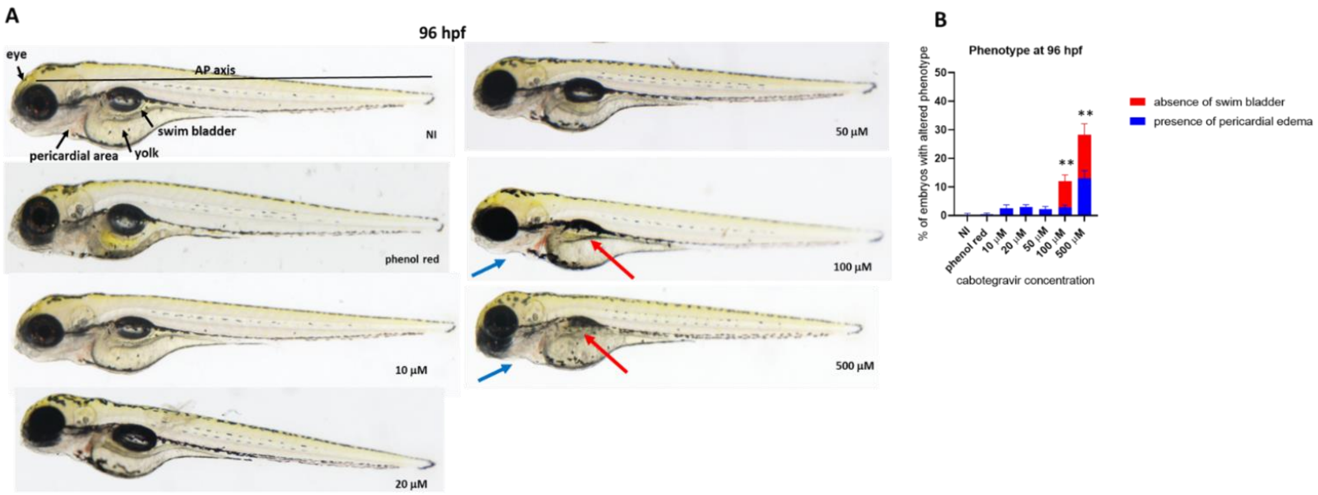

Supplementary Figure S3. Representative pictures at 96 hours post fertilization (hpf) of the gross morphological effects on zebrafish embryos after exposure to cabotegravir (CAB) directly microinjected at 1-2 cell stage. *(A)* CAB was dissolved in fish water containing 0.1% dimethyl sulfoxide (DMSO) at the same concentrations used for immersion experiments (10-20-50-100-500 µM). CAB solutions were co-injected with 0.05% phenol red as a tracer and control for microinjection process. Untreated control embryos were microinjected with fish water plus 0.1% DMSO plus 0.05% phenol red [phenol red]. Non-injected embryos were also used as controls [NI]. Total volume of injection was 5 nL/embryo. All treatments including controls were conducted 3 times, with 50 embryos for each experiment and each treatment. Embryos in the figures are representative of all experiments. Evaluation of gross morphology [body length, body weight, eye dimension, head and tail morphology, anterior-posterior (AP) axis, absence or presence of pericardial edema, absence or presence of the inflated swim bladder, yolk sac morphology and dimension] was performed on anesthetized embryos (0.4% Tricaine) under direct visualization with Zeiss Axiozoom V13 (Zeiss) microscope, equipped with PlanNeoFluar Z 1x/0.25 FWD 56 mm lens and Zen Pro software (magnification 20X). All pictures are lateral views with dorsal to the top and anterior to the left. The blue arrow indicates the absence of an inflated swim bladder, and the red arrow indicates the presence of pericardial edema in 100 and 500 µM CAB-treated embryos. *(B)* Percentages of embryos with defects. Results are expressed as mean ± SD of three independent experiments, with 30 embryos for each experiment and each treatment. Statistical analysis was performed with GraphPad Prism 8.3 Software by Student's t test. *p*-values less than 0.05 were considered as significant (* $p < 0.05$ vs. control group; ** $p < 0.005$ vs. control group).

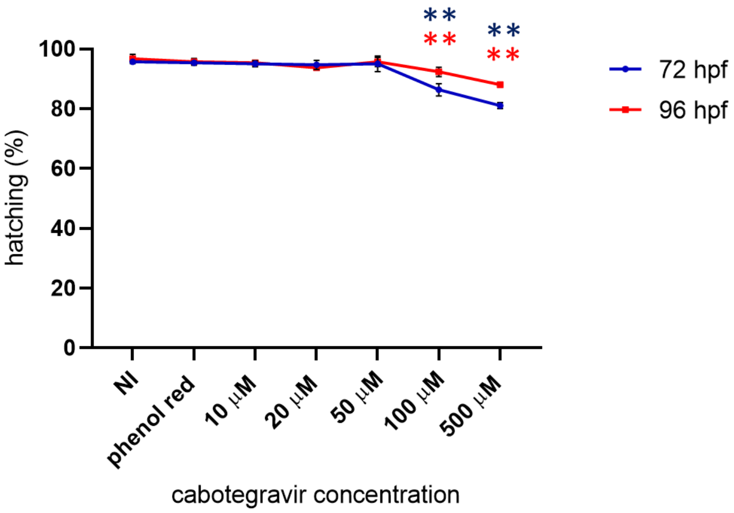

Supplementary Figure S4. Percentages of hatched embryos at 72 and 96 hours post fertilization (hpf) after exposure to cabotegravir (CAB) directly microinjected at 1-2 cell stage. CAB was dissolved in fish water containing 0.1% dimethyl sulfoxide (DMSO) at the same concentrations used for immersion experiments (10-20-50-100-500 µM). CAB solutions were co-injected with 0.05% phenol red as a tracer and control for microinjection process. Untreated control embryos were microinjected with fish water plus 0.1%, DMSO plus 0.05% phenol red [phenol red]. Non-injected embryos were also used as controls [NI]. Total volume of injection was 5 nL/embryo. X-axis shows drug doses used for exposure of embryos; Y-axis shows the corresponding percentages of hatching embryos at 72 (blue line) and 96 (red line) hpf. Results are expressed as mean ± SD of three independent experiments, with 50 embryos for each experiment and each treatment. Statistical analysis was performed with GraphPad Prism 8.3 Software by Student's t test. *p*-values less than 0.05 were considered as significant (* $p < 0.05$ vs. control group; ** $p < 0.005$ vs. control group).
